# Mapping of multiple criteria for priority setting of health interventions: an aid for decision makers

**DOI:** 10.1186/1472-6963-12-454

**Published:** 2012-12-13

**Authors:** Noor Tromp, Rob Baltussen

**Affiliations:** 1 Department of Primary and Community Care (117), Radboud University Nijmegen Medical Center (RUNMC). NICHE (Nijmegen International Center for Health Systems Research and Education), PO Box 9101, HB Nijmegen, 6500, The Netherlands

**Keywords:** Decision making, Priority setting, Multi-criteria decision analysis

## Abstract

**Background:**

In rationing decisions in health, many criteria like costs, effectiveness, equity and feasibility concerns play a role. These criteria stem from different disciplines that all aim to inform health care rationing decisions, but a single underlying concept that incorporates all criteria does not yet exist. Therefore, we aim to develop a conceptual mapping of criteria, based on the World Health Organization’s *Health Systems Performance* and *Health Systems Building Blocks* frameworks. This map can be an aid to decision makers to identify the relevant criteria for priority setting in their specific context.

**Methods:**

We made an inventory of all possible criteria for priority setting on the basis of literature review. We categorized the criteria according to both health system frameworks that spell out a country’s health system goals and input. We reason that the criteria that decision makers use in priority setting exercises are a direct manifestation of this.

**Results:**

Our map includes thirty-one criteria that are distributed among five categories that reflect the goals of a health system (i.e. to improve level of health, fair distribution of health, responsiveness, social & financial risk protection and efficiency) and leadership/governance one category that reflects feasibiliy based on the health system building blocks (i.e. service delivery, health care workforce , information, medical products, vaccines & technologies, financing and).

**Conclusions:**

This conceptual mapping of criteria, based on well-established health system frameworks, will further develop the field of priority setting by assisting decision makers in the identification of multiple criteria for selection of health interventions.

## Background

Concerns on the costs, effectiveness and cost-effectiveness of health interventions have dominated the debate on health rationing in a wide range of countries since long [1-3]. More recently, the explicit use of a number of equity-related criteria have been put forward, like severity of disease, socio-economic status, or gender, reflecting the increased attention for distribution of health in a population, as summarized by Johri and Norheim [4]. Still other criteria, like ease of implementation [5] or political acceptability [6] are presently finding their way in the prioritization of health interventions. The recognition that not a single but multiple criteria should explicitly be considered has led to the development of multi-criteria decision analysis (MCDA). This method sets programme priorities by referring to a comprehensive set of explicit criteria and guides decision makers in understanding the trade-offs between values that may be conflicting. For example while mobile clinics for HIV testing may be costly and therefore inefficient, they may deserve priority because they reach out to remote areas and therefore contribute to equity in service delivery. A core component in any MCDA is the identification of criteria that decision-makers consider important in their specific contexts. As a next step, MCDA scores the performance of health interventions on these criteria [7,8].

At the same time, surprisingly little work has been done to develop a meaningful conceptual mapping of criteria that can help to identify the relevant set of criteria. A recent report that advices the UK’s National Institute for Health and Clinical Excellence (NICE) for the use of MCDA reviewed the literature and one of the findings was that most applications have a fixed set of criteria and lack explanation of the rationale behind the selected criteria and the categories used. In addition, it was concluded that the applications give neither flexibility nor assistance to decision makers to select an unique set of criteria in their decision context [8]. More specifically, in 1999, Musgrove presented the ‘nine criteria for public spending on health care’ in a spider-web like diagram, however, without classification of criteria [9]. Baltussen and Niessen presented in 2006 the ‘cloud of criteria’, suggesting that criteria cannot be systematically categorized [7]. Another framework, introduced by EVIDEM in 2010, does not explain an underlying rationale for the choice of categories that are used [10,11]. Furthermore, various reviews simply list priority setting criteria [4,8,11,12]. Only the list of criteria reported in the review of Golan *et al.*, categorizes criteria according to the principles of allocative justice for rationing health care, i.e. need, maximizing and egalitarian principles, but is therefore limited in scope [13]. Our paper aims to develop a conceptual mapping of a comprehensive set of criteria, including efficiency, equity and feasibility concerns.

Categorization of criteria is important for two main reasons. Firstly, for decision makers, the grouping of a large and diffuse set of criteria into categories may ease their interpretation and facilitate decision-making. Second, such a categorization may be an aid to well-define criteria, and to avoid overlap and double-counting of criteria. Criteria, especially those related to health distribution (i.e. equity) concerns are often difficult to define and a proper mapping sets boundaries to facilitate this.

## Methods

### Conceptual mapping of criteria

**Figure 1:**
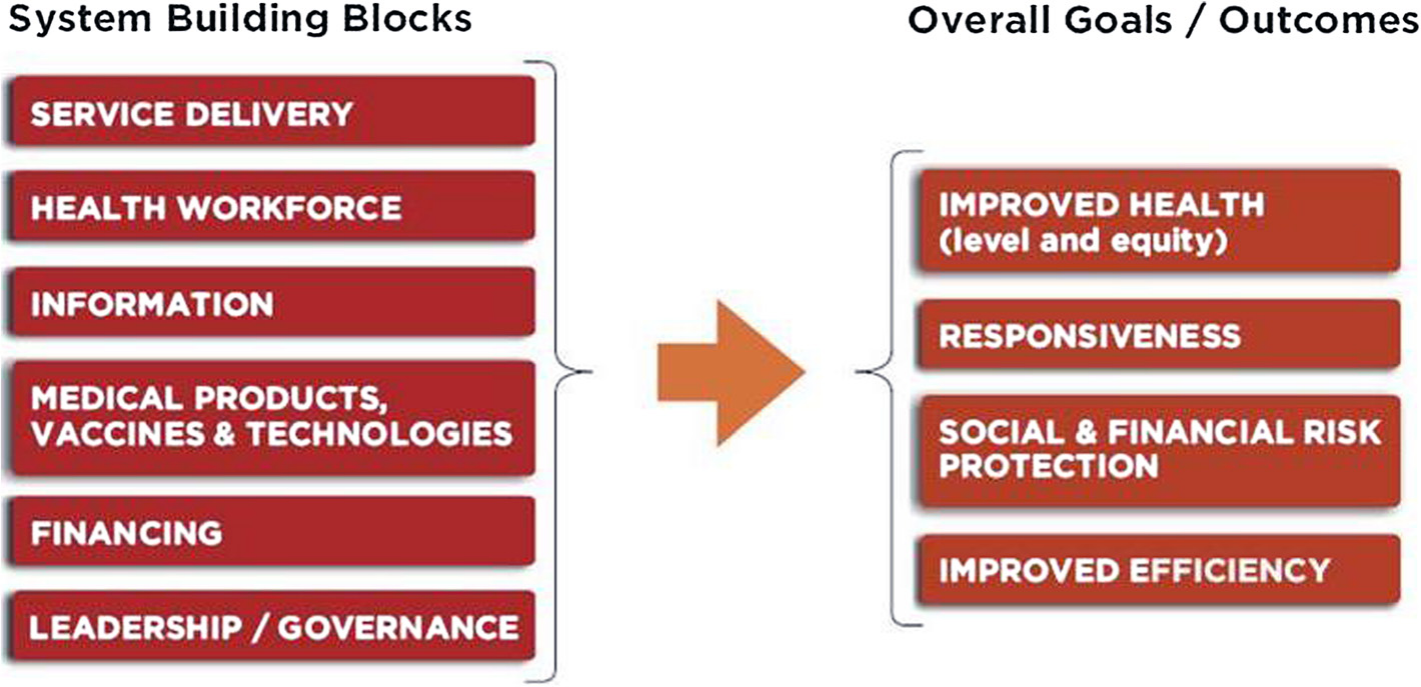
The building blocks and goals of the health system.

This paper introduces a conceptual mapping of criteria based on an integration of two well-established health systems frameworks, i.e. the World Health Organization’s (WHO) *Health Systems Performance framework*[14] and *Health Systems Building Blocks framework*[15,16] (Figure 1). These frameworks spell out a country’s health system goals and input – we reason that the criteria that decision makers use in priority setting exercises are a direct manifestation of this. More specifically, the *Health System Performance framework* is a generally accepted concept to reflect the goals of a health system. Here, in our interpretation, the framework reflects criteria that indicate the goals of interventions in health, i.e. to improve the level and distribution of health, to improve responsiveness, to offer financial protection and to make efficient use of resources. This can be loosely defined as ‘what *should* a health system do’. The *Health System Building Blocks framework* is a generally accepted concept to reflect the required components (or inputs) for an effective health system. Here, in our interpretation, the framework reflects criteria that relate to the feasibility of interventions, or loosely defined as ‘what *can* a health system do’. These criteria relate to the building blocks: ‘service delivery’, ‘health workforce’, ‘information’, ‘medical products, vaccines & technologies’, ‘financing’, and ‘leadership/governance’. Together, the two frameworks offer a comprehensive framework for classifying priority setting criteria. We employ both WHO frameworks because they are worldwide used by decision makers at country level and are credible conceptualizations of health systems [15,17].

We carried out two steps to develop our conceptual mapping of criteria. In a first step, we made an inventory of all possible criteria for priority setting on the basis of literature reviews [4,8,11-13]. In a second step, we categorized these criteria according to the health systems goals and building blocks, based on their definitions (Table 1). For example, following the health system performance framework, we distinguished the objective to improve health in two categories, i.e. to improve the ‘level of health’ and the ‘distribution of health’. We defined and classified criteria in these categories in order to avoid overlap between criteria.

**Table 1:** Definitions of categories used in the criteria map (based on the health system goals and building blocks)

***Category***	***Definition***
**Health system goals**[14]	
Health level	To improve the total average level of health in the population.
Health distribution	To achieve absence of avoidable or remediable differences in health among groups of people, defined socially, economically, demographically, or geographically.
Responsiveness	To use interventions that are responsive to people’s expectations in regard to non-health matters and reflect the importance of people’s dignity, autonomy and the confidentiality of information.
Social & financial risk protection	To provide financial protection against the costs of ill-health
Improved efficiency	To make the best and most efficient use of resources.
**Health system building blocks**[15]	
Service delivery	Good health services are those which deliver effective, safe, quality personal and non-personal health interventions to those that need them, when and where needed, with minimum waste of resources.
Health workforce	A well-performing health workforce is one that works in ways that are responsive, fair and efficient to achieve the best health outcomes possible, given available resources and circumstances (i.e. there is sufficient staff, fairly distributed; they are competent, responsive and productive).
Information	A well-functioning health information system is one that ensures the production, analysis, dissemination and use of reliable and timely information on health determinants, health system performance and health status.
Medical products, vaccines & technologies	A well-functioning health system ensures equitable access to essential medical products, vaccines and technologies of assured quality, safety, efficacy and cost-effectiveness, and their scientifically sound and cost-effective use.
Financing	A good health financing system raises adequate funds for health, in ways that ensure people can use needed services, and are protected from financial catastrophe or impoverishment associated with having to pay for them. It provides incentives for providers and users to be efficient.
Leadership/governance	Leadership and governance involves ensuring strategic policy frameworks exist and are combined with effective oversight, coalition-building, regulation, attention to system-design and accountability.

## Results

**Figure 2:**
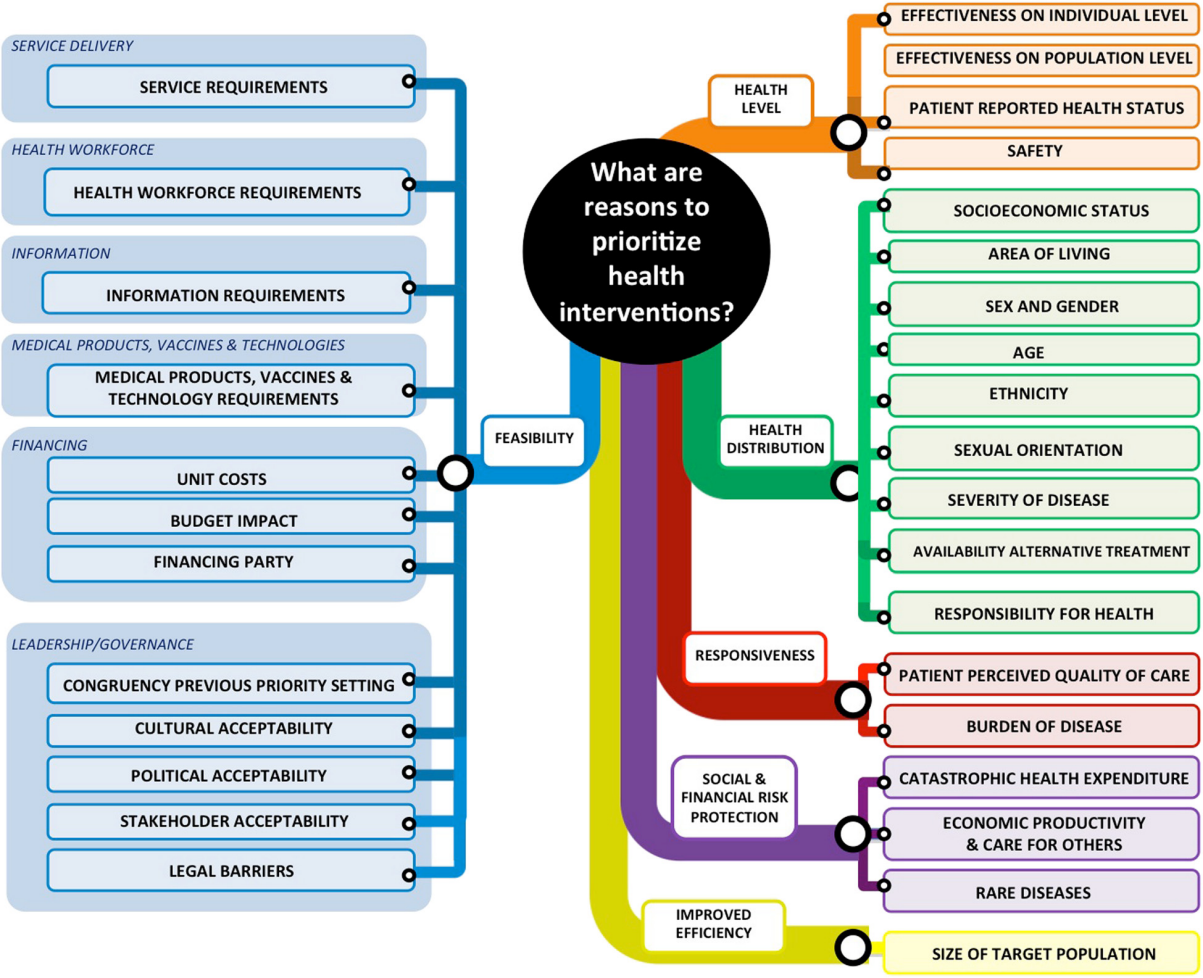
Mapping of priority setting criteria.

The conceptual mapping of criteria for priority setting is provided in Figure 2. The definitions of the categories are similar to those in the original WHO health system frameworks and are presented in Table 1. Our literature review resulted in the identification of a large set of criteria, which are often similar in concept but different in the ways they are described. In the Additional file 1, Overview of criteria considered for inclusion in criteria map, we list all criteria we considered for inclusion in our framework, and present a rationale for their inclusion or exclusion. The included thirty-one criteria are all single not-overlapping arguments to prioritize health interventions and are defined in Table 2. Here, we will give the rationale used for a selection of the criteria considered.

**Table 2:** Definitions of criteria for priority setting included in the criteria map

**Category**		**Criteria**	**Definition**
Health level		Effectiveness on individual level	Interventions that are effective in reduction of the morbidity and mortality, as measured on individual person level, may deserve priority.
		Effectiveness on population level	Interventions that are effective in reduction of the morbidity and mortality, as measured on population level, may deserve priority.
		Patient reported health status	Interventions that have high impact on patient reported health status may deserve priority.
		Safety	Interventions that do not harm in terms of morbidity and mortality may deserve priority.
Health distribution		Various criteria	All criteria proposed in the map have the same underlying rationale: all people should have as much of a fair chance to live a healthy life, and therefore interventions focusing on certain social groups may deserve priority.
Responsiveness		Patient perceived quality of care	Interventions that are responsive according to patient’s expectations of quality of care may deserve priority.
		Burden of disease	Interventions that focus on a high burden of disease in society may deserve priority.
Social & financial risk protection		Catastrophic health expenditure	Health care related costs can push people into poverty. Interventions that protect people against catastrophic health expenditure may deserve priority.
		Economic productivity & care for others	People who are economically productive and/or take care of others and become ill face income loss and health related costs, which could lead to poverty. Interventions that target those people may deserve priority.
		Rare diseases	Interventions for rare diseases might be very costly (because of the small number patients) and could push people into poverty. Therefore, these interventions may deserve priority.
Improved efficiency		Size of target population	Interventions that show economies of scale because they target a high number of people may deserve priority.
Feasibility	Service delivery	Service requirements	Interventions that are easy to implement because of the current service capacity may have priority. E.g. availability of: service infrastructure, delivery models, safety and quality and management.
	Health workforce	Health workforce requirements	Interventions that are easy to implement because of the current health workforce capacity may have priority. E.g. availability workforce and workforce policies, preferences of workforce for working conditions.
	Information	Information requirements	Interventions that are easy to implement because of the current information system capacity may have priority. E.g. availability of surveillance systems.
	Medical products, vaccines & technology	Medical products, vaccines & technology requirements	Interventions that are easy to implement because of the current medical products, vaccines & technology capacity may have priority. E.g. norms, standards and reliability procurement.
	Financing	Unit costs	Interventions that have small unit cost per patient may have priority.
		Budget impact	Interventions that consume a small part of the budget may have priority.
		Financing party	Interventions that receive sustainable financing may have priority.
	Leadership/governance	Congruency previous priority setting	Interventions that are in line with previous spending pattern may have priority.
		Cultural acceptability	Interventions that are cultural acceptable, because of the norms and values, may have priority.
		Political acceptability	Interventions that are political acceptable may have priority.
		Stakeholder acceptability	Interventions that are accepted by important stakeholder groups (e.g. patients groups, taxpayers, health care providers, donor agencies, voters) may have priority.
		Legal barriers	Interventions that face no legal barriers may have priority.

On the right panel of the map we distinguish five categories of criteria related to intervention’s goals. The first category is ‘health level’, and includes criteria ‘effectiveness on individual level’, ‘effectiveness on population level’ ‘patient reported health status’ and ‘safety’. Whereas reviews include ‘quality of evidence on effectiveness’, we excluded this from our map, as we consider quality of evidence to be relevant to all criteria, e.g. how costly or complex an intervention is. Rather, we propose to capture quality of evidence in uncertainty analysis. The second category is ‘health distribution’, and included criteria that relate to the core concept of ‘equal life time health’, which means that all people - independent of their background, their disease status or the availability of treatment - should have a fair chance to live a full healthy life [18]. This concept encompasses both horizontal and vertical equity. We define horizontal equity as the provision of *equal treatment for people with equal health needs.* Horizontal equity is non-discriminative towards certain groups in society to give them equal access to care as other groups with the same needs. We define vertical equity as the provision of *unequal but equitable treatment for people with unequal health needs* – this implies giving priority to certain groups in society, on the basis of their background (‘socioeconomic status’, ‘area of living’, ‘sex and gender’, ‘age’, ‘ethnicity’, ‘sexual orientation’), disease status (‘severity of disease’) or the ‘availability of treatment’ [19]. ‘Responsibility for health’ is included as interventions that focus on people that have bad luck may deserve more priority. The precise identification and definition of equity-related criteria is topic of a recent collaboration between different experts (ethicists, public health experts, economist, etc.) from academic institutes and the WHO [4,19]. Therefore we only provisionally list and define these criteria here.

The third category is ‘responsiveness’, and includes ‘patient perceived quality of care’. Interventions should be responsive to people’s expectations in regard to non-health matters and reflect the importance of people’s dignity and autonomy, and the confidentiality of information. Although this seems a general, and therefore system-wide concern, some interventions do better than other interventions in satisfying perceived quality of care. We include ‘burden of disease’ to represent the wish of society to target high burden diseases. The fourth category is ‘social and financial risk protection’ and includes ‘catastrophic health expenditure’, ‘economic productivity and care for others’ and ‘rare diseases’. Regarding the latter, interventions targeting rare diseases may deserve priority because they may be very costly (as intervention (especially drug) development costs are only shared by a small number of patients) and could push people into poverty. The fifth category is ‘improved efficiency’ and reflects the economies of scale that can be obtained when reaching large number of people. Therefore we have included the criterion ‘size of target group’. We decided to exclude ‘cost-effectiveness’ as a criterion on itself, as theoretically costs (as a feasibility constraint) and effectiveness (as a goal) are both implicitly included in the mapping as individual criteria. However, regarding the importance of the criterion of cost-effectiveness in decision-making, decision-makers may nevertheless consider it as a separate criterion.

On the left panel of the map we distinguish one category of criteria related to the feasibility of implementation of interventions. This category is divided in six subcategories, based on the building blocks, i.e. ‘service delivery’, ‘health workforce’, ‘information’, ‘medical products, vaccine and technologies’, ‘financing’, and ‘leadership/governance’. In contrast to the criteria related to intervention goals (discussed above), little work has been done on these criteria (except on the criteria ‘cost’) and the criteria we put forward are first propositions. The first four subcategories relate to the current capacity of the health system and criteria reflect the requirements the for implementation of an intervention. The fifth subcategory on financing encompasses ‘unit costs’, ‘budget impact’ and ‘financing party’. The ‘unit costs’ are the total costs per patient from a health systems perspective whereas 'budget impact' incorporates the scale of an intervention. The ‘financing party’ criterion captures who is paying for a health intervention and reflects notions on its financial sustainability. The sixth subcategory represents the feasibility in terms of leadership/governance and includes ‘congruency previous priority setting’, ‘cultural acceptability’, ‘political acceptability’, ‘stakeholder acceptability’, and ‘legal barriers’.

## Discussion

Our map should not be regarded as a top-down expert advice on a fixed set of criteria that should always be considered in all prioritization decisions, but rather as an aid to decision-makers in their selection of relevant criteria. We see two broad applications of priority setting, and therefore of our mapping of criteria. First, it can inform decision makers who work in a specific context, e.g. on the reimbursement decision of a single intervention. These decisions are taken in the presence of a known budget and are likely limited by factors such as the currently available physical infrastructure, human resources or political consideration, at least in the short- to medium-term. This is labeled ‘context-specific priority setting’. The second application is to guide decisions on a wide range of interventions, to provide general information on their relative rank order to arrive a more informed debate on resource allocation priorities. Because priority setting in this application is not meant to provide a solution to a specific resource allocation question, it need not be highly contextualized in terms of e.g. physical infrastructure and/or human resources constraints. This is labeled ‘generalized priority setting’ [20]. The set of criteria for ‘context-specific priority setting’ is likely to be much more specific than those for ‘generalized priority setting’, but stem from the same conceptual mapping of criteria as presented above. That our mapping of criteria should not be considered as a fixed set of criteria is especially clear when setting priorities in a specific disease area. For example, an important criterion in HIV/AIDS health rationing decisions is whether the intervention reduces stigma. To include these disease-specific criteria in a generic list would increase its total number of criteria and make it unmanageable.

As our mapping is based on the WHO health systems frameworks, it has a certain credibility among decision makers. However, the choice of a different underlying framework might lead to another mapping of criteria.

In our framework, we included criteria related to the health system inputs and health system goals, but no criteria related to intermediate outcome measures as access and utilization. These measures are instrumental to reach health system goals, and are as such no goals in themselves. However, decision-makers can use them to monitor and evaluate progress towards the realization of the health system goals.

Another important step in MCDA is to define indicators to operationalize the criteria. For example, the severity of disease that an intervention targets can be measured in terms of health state valuations [21] and health gains in terms of disability adjusted life years averted. The operationalization of criteria would complement our mapping of criteria, and would allow the construction of a performance matrix that systematically demonstrates the performance of an intervention on all criteria [7,8]. Such a matrix can consequently be the basis for rationing decisions on (a set of) health interventions. Such decisions should eventually also account for non-quantifiable criteria related to e.g. complicated ethical judgments. These criteria are not reflected in our framework, and further research should be carried out on how these can best be accounted for, e.g. through a process of elaboration [8,20,22].

We consider the presented mapping of priority setting criteria as preliminary only and not as a final map. We welcome discussions to further develop it, to improve the use of MCDA for setting priorities in health.

## Conclusions

This conceptual mapping of criteria, based on well-established health system frameworks, will further develop the field of priority setting by assisting decision makers in the identification of multiple criteria for selection of health interventions.

## Abbreviations

EVIDEM: Evidence and Value: Impact on DEcisionMaking; MCDA: Multi Criteria Decision Analysis; NICE: National Institute for Health and Clinical Excellence; WHO: World Health Organization.

## Competing interests

The authors declare that they have no competing interests.

## Authors’ contributions

NT contributed to the design of the study, led the data collection and selection of criteria for inclusion in the map and drafted and finished the article. RB contributed to the design of the study and the selection of criteria for inclusion in the map and assisted in writing the article.

## Pre-publication history

The pre-publication history for this paper can be accessed here:

http://www.biomedcentral.com/1472-6963/12/454/prepub

## Supplementary Material

Additional file 1 Overview of criteria considered for inclusion in criteria map. Click here for file

## References

[B1] Bank W World Development Report: Investing in Health 1993 New York: Oxford University Press

[B2] Ham C Priority setting in health care: learning from international experience Health policy (Amsterdam, Netherlands) 1997 42 1 49 66 10.1016/S0168-8510(97)00054-7 10173493

[B3] WHO-CHOICE Making choices in health: WHO guide to cost-effectiveness analysis 2003 Geneva: World Health Organization

[B4] Johri M Norheim O Can cost-effectiveness analysis integrate concerns for equity? systematic review Int J Technol Assess Health Care 2012 28 2 10.1017/S0266462312000050 22494637

[B5] Gericke CA Kurowski C Ranson MK Mills A Intervention complexity–a conceptual framework to inform priority-setting in health Bull World Health Org 2005 83 4 285 293 15868020 PMC2626218

[B6] Goddard M Hauck K Smith PC Priority setting in health - a political economy perspective Health Econ Policy Law 2006 1 Pt 1 79 90 18634704 10.1017/S1744133105001040

[B7] Baltussen R Niessen L Priority setting of health interventions: the need for multi-criteria decision analysis Cost Eff Resour Alloc 2006 4 14 10.1186/1478-7547-4-14 16923181 PMC1560167

[B8] Devlin N Sussex J Incorporating multiple criteria in HTA: methods and processes 2011 London: Office of Health Economics http://www.ohe.org/publications/article/incorporating-multiple-criteria-in-hta-methods-and-processes-8.cfm

[B9] Musgrove P Public spending on health care: how are different criteria related? Health policy (Amsterdam, Netherlands) 1999 47 3 207 223 10.1016/S0168-8510(99)00024-X 10538919

[B10] The EVIDEM (Evidence and Value: Impact on DEcisionMaking) frameworkhttp://www.evidem.org

[B11] Guindo L Wagner M Baltussen R Rindress D Van Til J Kind P Goetghebeur M From efficacy to equity: literature review of decision criteria for resource allocation and healthcare decisionmaking Cost Eff Resour Alloc 2012 10 9 10.1186/1478-7547-10-9 22808944 PMC3495194

[B12] Cleary SM Mooney GH McIntyre DE Claims on health care: a decision-making framework for equity, with application to treatment for HIV/AIDS in South Africa Health Policy Plan 2011 26 6 464 470 10.1093/heapol/czq081 21186205 PMC3199038

[B13] Golan O Hansen P Kaplan G Tal O Health technology prioritization: Which criteria for prioritizing new technologies and what are their relative weights? Health policy (Amsterdam, Netherlands) 2011 102 2–3 126 135 10.1016/j.healthpol.2010.10.012 21071107

[B14] World Health Organization The world health report 2000 - Health systems: improving performance 2000 Geneva: World Health Organization

[B15] World Health Organization Everybody’s Business: Strengthening Health Systems to Improve Health Outcomes: WHO’s Framework for Action 2007 Geneva: World Health Organization

[B16] World Health Organization The world health report 2008 - Primary health care: now more than ever 2008 Geneva: World Health Organization

[B17] Frenk J Bridging the divide: global lessons from evidence-based health policy in Mexico Lancet 2006 368 9539 954 961 10.1016/S0140-6736(06)69376-8 16962886

[B18] Daniels N Just Health 2008 Cambridge: Cambridge University Press

[B19] World Health Organization Setting equitable priorities in health and health care: from theory to practice Brocher Symposium 2011 2011 Geneva http://www.brocher.ch/pages/symppasses.asp

[B20] Baltussen R Youngkong S Paolucci F Niessen L Multi-criteria decision analysis to prioritize health interventions: Capitalizing on first experiences Health policy (Amsterdam, Netherlands) 2010 96 3 262 264 10.1016/j.healthpol.2010.01.009 20206403

[B21] Murray C Quantifying the burden of disease: the technical basis for disability-adjusted life years Bull World Health Org 1994 72 3 429 45 8062401 PMC2486718

[B22] Youngkong S Baltussen R Tantivess S Koolman X Teerawattananon Y Criteria for priority setting of HIV/AIDS interventions in Thailand: a discrete choice experiment BMC Heal Serv Res 2010 10 197 10.1186/1472-6963-10-197 PMC2912896 20609244

