# Supplementary material for: Mapping of multiple criteria for priority setting of health interventions: an aid for decision makers
Source: BMC Health Serv Res. 2012 Dec 13;12:454. doi: 10.1186/1472-6963-12-454 (PMC3565954; doi:10.1186/1472-6963-12-454)
Supplement: Additional file 1 — Overview of criteria considered for inclusion in criteria map. [file 1472-6963-12-454-S1.docx]

| **Appendix A. Overview of criteria considered for inclusion in criteria map** | | | |
| --- | --- | --- | --- |
| This appendix contains an overview of all criteria considered for inclusion in our map according to the consulted data sources. A rationale is given on why a criterion is excluded. For all the criteria that are included, we present the category and criteria it belongs to and for the related definitions we refer to table 1 and 2 of the article. | | | |
| Table 1. Overview of criteria presented by review of Guindo et al 2012 | | | |
| ***Criteria category*** | ***Criteria*** | ***Excluded/ Included (category/criterion)*** | ***Rationale for exclusion*** |
| A-Health outcomes and benefits of intervention | A1: Health benefit | Included (health level, effectiveness on individual level) |  |
|  | A2: Efficacy/effectiveness | Included (health level, effectiveness on individual level and effectiveness on population level) |  |
|  | A3: Life saving | Included (health level, effectiveness on individual level) |  |
|  | A4: Safety | Included (health level, safety) |  |
|  | A5: PRO (patient reported outcomes) | Included (health level, patient reported outcomes) |  |
|  | A6: Quality of care | Included (responsiveness, patient reported quality of live) |  |
| B-Type of health benefit | B1: Population effect (prevention) | Included (health level, effectiveness on individual level and effectiveness on population level) |  |
|  | B2: Individual effect (medical service) | Included (health level, effectiveness on individual level) |  |
| C-Impact of the disease targeted by intervention | C1: Disease severity | Included (health distribution, disease severity) |  |
|  | C2: Disease determinants | Included (health distribution, all criteria) |  |
|  | C3: Disease burden | Included (responsiveness, burden of disease) |  |
|  | C4: Epidemiology | Included (responsiveness, burden of disease) |  |
| D-Therapeutic context of intervention | D1: Treatment alternatives | Included (health distribution, availability alternative treatment) |  |
|  | D2: Need | Included (improved efficiency, size of target population) |  |
|  | D3: Clinical guidelines & Practices | Included (feasibility, service requirements) |  |
|  | D4: Pre-existing use | Included (feasibility, congruency previous priority setting) |  |
| E-Economic impact of intervention | E1: Cost | Included (feasibility, unit costs) |  |
|  | E2: Budget impact | Included (feasibility, budget impact) |  |
|  | E3: Broad financial impact | Included (social & financial risk protection, economic productivity and care for others) |  |
|  | E4: Poverty reduction | Included (social & financial risk protection, catastrophic health expenditure) |  |
|  | E5: Cost-effectiveness | Implicitly included (this criterion is a combination of unit costs (financing building block) and effectiveness on individual/population level (health level goal)) |  |
|  | E6: Value | Included (health level, all criteria) |  |
|  | E7: Efficiency and opportunity costs | Efficiency is implicitly included (this criterion is a combination of unit costs (financing building block) and effectiveness on individual/population level (health level goal)), opportunity costs is included (feasibility, unit costs) |  |
|  | E8: Resources | Included (feasibility, all criteria) |  |
|  | E9: Insurance premiums | Excluded | This criterion is conceptually not linked to building blocks or goals. |
| F-Quality and uncertainty of evidence | F1: Evidence available | Excluded | The level of evidence can be captured in sensitivity analysis. |
|  | F2: Strength of evidence | Excluded | The strength of evidence can be captured in sensitivity analysis. |
|  | F3: Relevance of evidence | Excluded | Any evidence used in decision-making should be relevant and this differentiates between interventions. |
|  | F4: Evidence characteristics | Excluded | All relevant evidence characteristics should be included in decision-making and this differentiates between interventions. |
|  | F5: Research ethics | Excluded | This criterion is conceptually not linked to building blocks or goals. |
|  | F6: Evidence requirements | Excluded | Evidence is required in decision making on all criteria. |
| G- Implementation complexity of intervention | G1: Legislation | Included (feasibility, legal barriers) |  |
|  | G2: Organizational requirements and capacity to implement | Included (feasibility, service and health care workforce requirements) |  |
|  | G3: Skills | Included (feasibility, health care workforce requirements) |  |
|  | G4: Flexibility of implementation | Included (feasibility, service requirements) |  |
|  | G5: Characteristics of intervention | Included (all criteria) |  |
|  | G6: Appropriate use | Included (health level, safety) |  |
|  | G7: Barriers and acceptability | Included (feasibility, cultural, political and stakeholder acceptability) |  |
|  | G8: Integration and system efficiencies | Included (feasibility, all criteria) |  |
|  | G9: Sustainability | Included (feasibility, financing party) |  |
| H-Priorities fairness and ethics | H1 Population priorities | Included (health distribution, all criteria) |  |
|  | H2: Access | Excluded | This criterion is an intermediate outcome measure and is not directly related to the goals or building blocks. |
|  | H3: Vulnerable and needy population | Included (health distribution, all criteria) |  |
|  | H4: Equity, fairness and justice | Included (health distribution, all criteria) |  |
|  | H5: Utility | Included (all goals) |  |
|  | H6: Solidarity | Included (health distribution, all criteria) |  |
|  | H7: Ethics and moral aspects | Included (health distribution, all criteria) |  |
| I-Overall context | I1: Mission and mandate of health system | Included (all goals) |  |
|  | I2: Overall priorities | Included (feasibility, political acceptability/ all goals) |  |
|  | I3: Financial constraints | Included (feasibility, financing party) |  |
|  | I4: Incentives | Included (feasibility, stakeholder acceptability) |  |
|  | I5: Political aspects: | Included (feasibility, political acceptability) |  |
|  | I6: Historical aspects | Included (feasibility, congruency previous priority setting) |  |
|  | I7: Cultural aspects | Included (feasibility, cultural acceptability) |  |
|  | I8: Innovation | Included (medical products, vaccine and technology requirements) |  |
|  | I9: Partnership and leadership | Included (feasibility, political acceptability) |  |
|  | I10: Citizen involvement | Included (feasibility, stakeholder acceptability) |  |
|  | I11: Stakeholders interests and pressures | Included (feasibility, stakeholder acceptability) |  |

| **Table 2. Criteria included in EVIDEM framework (www.evidem.org)** | | |
| --- | --- | --- |
| ***Categories*** | | ***Criteria (overlap with criteria from Guindo review presented in table 1 above)*** |
| *MCDA model criteria (intrinsic criteria, universally operationalizable* | *Disease impact* | D1 - Disease severity 🡪 C1 in Table 1 |
|  |  | D2 - Size of population 🡪 D4 |
|  | *Context of intervention* | C1 - Clinical guidelines 🡪 D3 |
|  |  | C2 - Comparative interventions limitations *(unmet needs) 🡪 D2* |
|  | *Intervention outcomes* | I1 - Improvement of efficacy/ effectiveness 🡪 A2 |
|  |  | I2 - Improvement of safety & tolerability 🡪 A4 |
|  |  | I3 - Improvement of patient reported outcomes 🡪 A5 |
|  | *Type of benefit* | T1 - Public health interest *(e.g., prevention, risk reduction) 🡪 B1* |
|  |  | T2 - Type of medical service *(e.g., cure, symptom relief) 🡪 B2* |
|  | *Economics* | E1 - Budget impact on health plan *(cost of intervention) 🡪 E2* |
|  |  | E2 - Cost-effectiveness of intervention (optional) 🡪 E5 |
|  |  | E3 - Impact on other spending *(e.g., hospitalization, disability)* 🡪E3 |
|  | *Quality of evidence* | Q1 - Adherence to requirements of decisionmaking body 🡪 F6 |
|  |  | Q2 - Completeness and consistency of reporting evidence 🡪 F2 |
|  |  | Q3 - Relevance and validity of evidence 🡪 F2 |
| *Contextual (extrinsic) criteria* | *Ethical framework* | Et1 - Utility - Goals of healthcare 🡪H5 |
|  |  | Et2 - Efficiency - Opportunity costs 🡪 E7 |
|  |  | Et3 – Fairness - Population priority & access 🡪 H4 |
|  | *Contextual criteria* | O1 - System capacity & appropriate use of intervention 🡪 G2 |
|  |  | O2 - Stakeholder pressures 🡪 I11 |
|  |  | O3 - Political/historical context 🡪 I5/I6 |
| Note: In the last column we show the overlap between the criteria from evidem.org and Guindo et al (presented in table 1) | | |

| **Table 3. Criteria for equity presented by Johri & Norheim 2012** | | | |
| --- | --- | --- | --- |
| ***Category*** | ***Sample criteria*** | ***Inclusion/exclusion/represented by other criteria*** | ***Rationale for exclusion*** |
| Disease-related criteria | Disease severity | Included (health distribution, disease severity) |  |
|  | Poor capacity to benefit from treatment | Included (health distribution, disease severity) |  |
|  | Rare diseases | Included (social & financial risk protection, rare diseases) |  |
| Criteria related to characteristics of social groups | Equality among the members of distinct groups in relation to a normative standard of equal lifetime health. | Included (health distribution, all criteria) |  |
| Criteria related to protection against the financial and social effects of ill health | Economic productivity | Included (social & financial risk protection, economic productivity and care for others) |  |
|  | Catastrophic health expenditures | Included (social & financial risk protection, catastrophic health expenditures) |  |
|  | Impact on dependents | Included (social & financial risk protection, economic productivity and care for others) |  |
| Other | Personal responsibility for health | Included (health distribution, responsibility for health) |  |
|  | Discounting | Excluded | This is presented in Johri & Norheim in reaction to cost-effectiveness analysis. This criterion is conceptually not linked to building blocks or goals. |

| **Table 4. Criteria presented by Golan et al 2010** | | | |
| --- | --- | --- | --- |
| ***Principle of allocative justice*** | ***Criteria*** | ***Inclusion/exclusion/represented by other criteria*** | ***Rationale for exclusion*** |
| *Need* | General | Included (responsiveness, burden of disease) |  |
|  | Severity of the condition | Inclusion (health distribution, severity of disease) |  |
|  | Availability of alternatives | Inclusion (health distribution, availability alternative treatment) |  |
| *Appropriateness* | Efficacy and safety | Included (health level, effectiveness on individual level, effectiveness on population level, safety) |  |
|  | Effectiveness | Included (health level, effectiveness on individual level) |  |
| *Clinical benefits* | General | Included (health level, effectiveness on individual level) |  |
|  | Effect on mortality (life-saving) | Included (health level, effectiveness on individual level) |  |
|  | Effect on longevity | Included (health level, effectiveness on individual level) |  |
|  | Effect on health-related-quality-of-life | Included (health level, effectiveness on individual level) |  |
| *Efficiency* | Cost-effectiveness/benefit | Implicitly included (this criterion is a combination of unit costs (financing building block) and effectiveness on individual/population level (health level goal)) |  |
|  | Budgetary impact | Included (feasibility, budget impact) |  |
|  | Cost | Included (feasibility, unit costs) |  |
| *Equality* | General | Included (health distribution, all criteria) |  |
|  | Accessibility to the service | Excluded | This criterion is an intermediate outcome measure and therefor indirectly related to the goals and building blocks of our map. |
|  | Affordability to the individual | Included (health distribution, socio economic status/social and financial risk protection, catastrophic health expenditure) |  |
| *Solidarity* | - |  |  |
| Other ethical or social values | Autonomy | Included (responsiveness, patient perceived quality of care) |  |
|  | Public health value | Included (all goals) |  |
|  | Impact on future generations | Included (health level, effectiveness on population level) |  |
| Quality of the clinical and economic evidence **(‘Other considerations’)** | - | Excluded | The quality of the clinical and economic evidence can be captured in sensitivity analysis. |
| Other considerations not elsewhere classified **(‘Other considerations’)** | Strategic issues consistency with previous decisions and precedents | Inclusion (feasibility, congruency previous priority setting) |  |

| **Table 5. Criteria presented by Devlin et al 2010 (additional to Golan et al 2010)** | | | |
| --- | --- | --- | --- |
| NICE (England) – factors taken into account in judgements about cost effectiveness | | | |
| ***Criteria*** | ***Inclusion/exclusion/represented by other criteria*** | | ***Rationale for exclusion*** |
| Severity of underlying illness | Included (health distribution, severity of disease) | |  |
| End of life treatments | Included (health distribution, severity of disease) | |  |
| Stakeholder persuasion | Included (feasibility, stakeholder acceptability) | |  |
| Significant innovation | Included (feasibility, medical products, vaccines and technology requirements) | |  |
| Disadvantaged populations | Included (health distribution, all criteria) | |  |
| Children | Included (health distribution, age) | |  |
| AGNSS 2010 (Advisory Group for National Specialised Service, England) 2010 | | | |
| ***Criteria*** | ***Inclusion/exclusion/represented by other criteria*** | | ***Rationale for exclusion*** |
| Health gain |  | |  |
| Severity and ability of patients to benefit | Included (health distribution, severity of disease) | |  |
| Clinical safety and risk | Included (health level, safety) | |  |
| Clinical effectiveness and potential for improving health | Included (health level, effectiveness on individual level) | |  |
| Societal value |  | |  |
| Stimulating research and innovation | Included (feasibility, medical products, vaccines and technology requirements) | |  |
| Needs of patients and society | Included (health distribution, severity of disease) | |  |
| Reasonable cost |  | |  |
| Average cost per client | Included (feasibility, unit costs) | |  |
| Overall cost impact and affordability including opportunity cost | Included (feasibility, budget impact / social & financial risk protection, catastrophic health expenditure) | |  |
| Value for money compared to alternatives | Implicitly included (this criterion is a combination of unit costs (financing building block) and effectiveness on individual/population level (health level goal)) | |  |
| Best practice |  | |  |
| Best clinical practice in delivering the service | Included (health level, effectiveness on individual level) | |  |
| Economic efficiency of provision | Implicitly included (this criterion is a combination of unit costs (financing building block) and effectiveness on individual/population level (health level goal)) | |  |
| Continuity of provision | Included (feasibility, financing party) | |  |
| Accessibility and balanced geographic distribution | Accessibility is excluded, balanced geographic distribution is included (health distribution, area of living) | |  |
| Huntingdonshire Primary Care Trust (Sub-national NHS commissioning, used MCDA on prioritization of their spending) (Box 5.1) | | | |
| ***Criteria*** | ***Inclusion/exclusion/represented by other criteria*** | ***Rationale for exclusion*** | |
| Effectiveness (QALYs) | Included (health level, effectiveness on individual level) |  | |
| Burden of disease | Included (responsiveness, burden of disease) |  | |
| Equity/fairness between social groups | Included (health distribution, all criteria) |  | |
| Deliverability and speed of implementation | Included (feasibility, all criteria) |  | |
| Engagement of public and professionals in demand management | Included (feasibility, health care workforce requirements) |  | |
| Acceptability to public and professions | Included (feasibility, cultural and stakeholder acceptability) |  | |
| Certainty/quality of evidence | Excluded | The certainty/quality of evidence can be captured in sensitivity analysis. | |
| Fit with national standards/targets | Included (feasibility, political acceptability) |  | |
| Isle of Wight Primary Care Trust (MCDA for commissioning Strategy 2008-2013) (Box 5.2) | | | |
| ***Criteria*** | ***Inclusion/exclusion/represented by other criteria*** | ***Rationale for exclusion*** | |
| Health benefit (= QALYs) | Inclusion (health level, effectiveness on individual level) |  | |
| Health inequalities between geographical areas, sexes, ‘special groups’ | Inclusion (health distribution, all criteria) |  | |
| Probability of success, comprising: ease of implementation, availability of workforce, acceptability to stakeholders, process complexity (number of steps needed) | Included (feasibility, all criteria) |  | |
| “Health Authority D” prioritising developments for use of additional funding  over the next five years using seven criteria. (NHS sub commission) | | | |
| ***Criteria*** | ***Inclusion/exclusion/represented by other criteria*** | ***Rationale for exclusion*** | |
| Evidence of effectiveness | Excluded | The evidence of effectiveness may be used to conduct a sensitivity analysis. | |
| Value for money | Implicitly included (this criterion is a combination of unit costs (financing building block) and effectiveness on individual/population level (health level goal)) |  | |
| Health gain or maintenance | Included (health level, effectiveness on individual level and effectiveness on population level) |  | |
| Equity | Included (health distribution, all criteria) |  | |
| Risk management | Excluded | This criterion is conceptually not linked to building blocks or goals. | |
| National or Board priority | Included (health distribution, political acceptability) |  | |
| Public preference | Included (feasibility, stakeholder acceptability) |  | |
| Argyll and Clyde Health Board in Scotland | | | |
| ***Criteria*** | ***Inclusion/exclusion/represented by other criteria*** | ***Rationale for exclusion*** | |
| Potential health gain | Included (health level, effectiveness on individual level/ effectiveness on population level) |  | |
| Prevention of ill health | Included (health level, effectiveness on individual level/ effectiveness on population level) |  | |
| Quality of life | Included (health distribution, severity of disease) |  | |
| Equity of access | Included (health distribution, all criteria) |  | |
| Addressing health status inequalities at population level | Included (health distribution, all criteria) |  | |
| Expressed demand | Included (feasibility, stakeholder acceptability) |  | |
| Appropriateness | Included (responsiveness, patient perceived quality of care) |  | |
| Strength of evidence | Excluded | The strength of evidence can be captured in sensitivity analysis. | |
| Known priorities | Included (feasibility, congruency previous priority setting) |  | |
| Department of health - Strategic Outline Case for Epsom General Hospital  redevelopment scheme (Box 5.3) | | | |
| ***Criteria*** | ***Inclusion/exclusion/represented by other criteria*** | ***Rationale for exclusion*** | |
| Patient safety | Included (health level, safety) |  | |
| Meet quality standards | Included (health level, safety) |  | |
| Performance + outcomes | Excluded | This criterion is not related to one specific goal or building blocks. In a decision making process the performance and outcomes on all relevant criteria should be taken into account. | |
| Long term clinical and financial stability | Included (feasibility, financing party) |  | |
| Productivity and efficiency of care services | Productivity is included (feasibility, service requirements) and efficiency is implicitly included (this criterion is a combination of unit costs (financing building block) and effectiveness on individual/population level (health level goal)) |  | |
| Patient focus | Included (responsiveness, patient perceived quality of care) |  | |
| Scope for modernization and innovation | Included (feasibility, medical products, vaccines and technology requirements) |  | |
| Achievability | Included (feasibility, all criteria) |  | |
| Strategic fit | Included (feasibility, political acceptability) |  | |
| Co-location of services | Included (feasibility, service requirement) |  | |
| Fits with “Centres of Clinical Excellence” | Included (feasibility, leadership) |  | |
| Fit with organizational cultures | Included (feasibility, leadership) |  | |
| Stakeholder’ expectations | Included (feasibility, stakeholder acceptability) |  | |
| Utilisation of estate | Included (feasibility, legal barriers) |  | |
| Department of health - Outline Business Case for the Royal National Orthopaedic Hospital,  Stanmore, redevelopment scheme (Box 5.4) | | | |
| ***Criteria*** | ***Inclusion/exclusion/represented by other criteria*** | ***Rationale for exclusion*** | |
| Centre of excellence | Included (feasibility, leadership) |  | |
| Quality of clinical care | Included (responsiveness, patients perceived quality of care) |  | |
| Patient centered | Included (responsiveness, patients perceived quality of care) |  | |
| Access | Excluded | This criterion is an intermediate outcome measure and is not directly related to the goals or building blocks. | |
| Elective capacity | Included (feasibility, political acceptability) |  | |
| Workforce recruitment and retention | Included (feasibility, health care workforce requirements) |  | |
| Suitable for working with partners | Included (feasibility, service requirements) |  | |
| Deliverability/achievability | Included (feasibility, all criteria) |  | |
| Design, sustainability and quality of life | Design is included (feasibility, all criteria), sustainability is included (feasibility, financing party), quality of life is included (health distribution, severity of disease) |  | |
| What impact does the patient’s condition have on their QoL | Included (health distribution, severity of disease) |  | |
| To what extent is the patient’s condition able to be ameliorated by treatment? | Included (health level, effectiveness on individual level) |  | |
| Israel’s Health Basket Committee: Pilot (Box 5.8) | | | |
| ***Criteria*** | ***Inclusion/exclusion/represented by other criteria*** | ***Rationale for exclusion*** | |
| Indicators: |  |  | |
| Lives saved, Life prolongation, Quality of life benefits | Included (health level, effectiveness on individual level) |  | |
| Availability of alternative treatments | Included (health distribution, availability alternative treatment) |  | |
| Other ethical/social benefits (e.g. reduces health gaps). | Included (health distribution, all criteria) |  | |
| Criteria: |  |  | |
| A. Benefits | Included (all goals) |  | |
| B. Net costs | Included (feasibility, unit costs) |  | |
| C. Quality of Evidence | Excluded | The quality of evidence may be used to conduct a sensitivity analysis. | |
| D. Other considerations | Included (all goals) |  | |
| Also presented on this case: |  |  | |
| pClEff - Probability that the new technology is clinically effective | Excluded | The probability that the new technology is clinically effective may be used to conduct a sensitivity analysis. | |
| pCostEf 20k - Probability that the new technology is cost-effective relative to the comparator at a willingness to pay of < 20k per QALY gained | Excluded | The probability that the new technology is cost-effective relative to the comparator at a willingness to pay of < 20k per QALY gained may be used to conduct a sensitivity analysis. | |
| Acceptability/Appropriateness/Preferences (of public and patients) | Included (feasibility, stakeholders acceptability) |  | |
| Terminality - End of life use | Included (health distribution, severity of disease) |  | |
| Orph/no alt/rescue - The new technology is an ‘orphan drug’ or it has no alternatives besides best supportive case, or it is used in a ‘rule of rescue’ situation. | Included (financial and social risk protection, rare diseases) |  | |
| OtherEq - Other equity considerations | Included (health distribution, all criteria) |  | |
| DH priorities - Clinical priority areas as designed by Secretary of State for Health and Welsh Assembly Government | Included (feasibility, leadership) |  | |
| Health System (HS) feasibility/impact (no additional definition provided) | Included (feasibility, all criteria) |  | |
| Innovativeness - (no additional definition provided) | Included (feasibility, medical products, vaccines and technology requirements) |  | |
| Wider Societal Considerations (no additional definition provided) | Included (social and financial risk protection/economic productivity and care for others) |  | |
| Figure 7.1 A template for explicit and transparent consideration of social value judgements in NICE’s deliberative process | | | |
| ***Criteria*** | ***Inclusion/exclusion/represented by other criteria*** | ***Rationale for exclusion*** | |
| End of life | Included (health distribution, severity of disease) |  | |
| Severity | Included (health distribution, severity of disease) |  | |
| Children | Included (health distribution, age) |  | |
| Social disadvantage | Included (health distribution, all criteria) |  | |
| Small patient numbers | Included (social & financial risk protection, rare diseases) |  | |
| Lack of alternative treatments | Included (health distribution, availability of alternative treatment) |  | |
| Aspects of innovation not taken into account the ICER | Innovation (feasibility, medical products, vaccines and technology requirements) |  | |
| Table 7.2 Attributes (criteria) and levels (the way that criteria are measured) included in a discrete choice experiment with NICE appraisal committee member | | | |
| ***Criteria*** | ***Inclusion/exclusion/represented by other criteria*** | ***Rationale for exclusion*** | |
| Incremental cost-effectiveness analysis | Implicitly included (this criterion is a combination of unit costs (financing building block) and effectiveness on individual/population level (health level goal)) |  | |
| Uncertainty | Excluded | The uncertainty of evidence can be captured in sensitivity analysis. | |
| Age | Included (health distribution, age) |  | |
| Baseline HR-QoL | Included (health distribution, severity of disease) |  | |
| Availability of other therapies | Included (health distribution, availability alternative treatment) |  | |

| **Table 6. Criteria (claims) presented by Cleary 2010 for considering to whom good should be distributed** | | |
| --- | --- | --- |
| ***Criteria*** | ***Inclusion/exclusion/represented by other criteria*** | ***Rationale for exclusion*** |
| Claim based on need as illness | Included (health distribution, all criteria) |  |
| Claim based on need as capacity to benefit | Included (health distribution, severity of disease) |  |
| Claim based on morally arbitrary bad luck | Included (health distribution, responsibility for health) |  |
| Claim based on deprivation or disadvantage | Included (health distribution, all criteria) |  |
| Claim based on extent to which someone is responsible for her HIV status | Included (health distribution, responsibility for health) |  |
| Claim based on the impact of treatment on social fabric | Included (social & financial risk protection, economic productivity and care for others) |  |
| Claim based on net impact on the health of society | Included (health level, effectiveness on population level) |  |
